# A Comparison with Civil Practice

**Published:** 1919-09-06

**Authors:** 


### THE ROYAL ARMY MEDICAL CORPS.

### A Comparison with Civil Practice.

ALTHOUGH it is now almost ten months since the Armistice, and the League of Nations is all but an accomplished fact, it is still quite uncertain how large must be the armed forces that the British Empire will be constrained to keep up; and, more particularly for our present purpose, what the size and condition is to be of the Army Medical Service. Those temporary and special reserve officers, therefore, who have lately been accepting commissions in the Royal Army Medical Corps must be doing so in some uncertainty what their prospects for the future are to be. It is clear enough that we must have an Army; no League of Nations can relieve us of the duty of policing the enormous tracts of the earth's surface where the *pax Britannica* is the only safeguard against battle, murder, and sudden death, to say nothing of pestilence and famine. That army must have a sufficient Medical Service; and to get this, Governments will have to understand that liberal conditions of employment for its commissioned officers are absolutely necessary.

There are scores of avenues in civil life opening up daily to tempt the man to whom a fixed salary—if a modest one—and a pension at the end of his service appeal more than the competitive hurly-burly of general or consulting practice. So the authorities have more and more to reckon with civilian services, as well as with the Indian Medical Service and the Medical Service of the Royal Navy, as competitors in the medical labour market. With all this increase of demand there is no immediate probability of increase of supply. The time during which a man is not earning any money whilst preparing for qualification is long and lengthening. The capital necessary to acquire qualification is far more now than twenty years ago, and likely to be greater still; the work necessary to gain a good knowledge of modern medicine and surgery is immense, is increasing, is a strain on the strongest, and the reward of the doctor is small when set against the time, money, and work he expended in his youth. There seems, therefore, to be an increased and increasing demand and a probability of a decreasing supply. Such conditions lead to a rise of price, and therefore qualified medical men will be able, if they choose, to obtain a higher money return for their services in the future than they have received in the past in civil life or in Army service. All these considerations make it probable that the officers of the R.A.M.C. will in future receive a higher rate of pay than they do at present, and even now, though the pay is not great, the advantages of the Service are many.

[NOTE.—The rates on which the following is based are the present ones—i.e., those of the 1914 Warrant. A new Warrant is due to be issued, increasing all the rates, that for a lieutenant being increased from 14s. to 20s. a day, and other ranks in proportion.]

The present pay does not look very tempting. To receive £700 a year at the age of forty-five years or over and £800 a year at fifty or so does not seem a big reward for perhaps six or seven years' study of a most exacting kind and the expenditure of probably at least £1,200 in acquiring a profession.

It is not a great reward, but it must be remembered that against that can be set certainty and no professional expenses. It is nett. It is what a man has in his hand to keep himself and his wife and family. The £470 of a captain of thirty-five years is equal to a practice of £700 a year, and, beyond pay and allowances, men who work hard and are keen can almost always get some extra pay, such as £50 a year specialist's pay in the junior ranks or charge pay in the senior ranks.

But the two great pecuniary advantages are the certain income and the certain pension. After twenty years a man can retire with £365 a year, and if he stays longer he can get a better pension still, about £540 after thirty years' service and £730 to a surgeon-general of sixty years of age. The money equivalent of such annuities is about £4,500. Besides the officers' own pensions there are pensions for wives and children, all of which advantages can be expressed in cash. The Pay Warrant gives the full particulars. When all these facts are considered a man is not far wrong if he concludes that he will be doing as well in the Army as he would have done in civil life if he had bought a practice of £500 a year, worked it up to £2,000, sold it for two years' purchase at sixty, and retired on an annuity bought with the proceeds and a few hundreds of savings. A nearer comparison of the pecuniary position of the civilian and the Army doctor is scarcely possible. The comparison will not appeal to the ambitious of a fortune; but it will to many who wish to live life.

When from his retirement a man looks back on his career, and compares it with the lot of those who were young with him, unless he be in poverty, he does not think only of the money he might have made had he done as this or that friend of his youth, but more of the time he has passed, of what he has seen, of what he has done, and of the lights and shades of the long vista back down which he looks. The Army doctor, if he has used his time wisely, should look back with pleasure and satisfaction. He will recall pleasant companions, sterling comrades, jolly days when he lived in this mess or that, scenes of travel, days of sport. Nor will he want for memories of strenuous times, of successful operations, of good work done, of grateful patients, of epidemics, of cholera camps, of fighting, wounds and death. There must be many men in the neighbourhood of Cavendish Square near to the summit of the ambition with which they left the examination halls who, when they talk with men of their year who have thrown in their lot with the Army, wonder if, after all, despite his smaller cash takings and his comparative obscurity, the old friend who has lived so wandering a life has not really had the best of it. Still more must this be so of him who has made a fair practice in some great factory town or tourist-ridden watering-place. Is the constant rush and round from this patient to that through the same dull familiar streets and the break of a fortnight's yearly holiday as satisfactory to look back on as another life that might have been? Who knows! We all have our regrets and our compensations.

